# Synthesis and HRTEM Investigation of EuRbFe_4_As_4_ Superconductor

**DOI:** 10.3390/nano12213801

**Published:** 2022-10-28

**Authors:** Alena Yu. Degtyarenko, Igor A. Karateev, Alexey V. Ovcharov, Vladimir A. Vlasenko, Kirill S. Pervakov

**Affiliations:** 1 V.L. Ginzburg Centre for High-Temperature Superconductivity and Quantum Materials, P.N. Lebedev Physical Institute of the Russian Academy of Sciences, 53, Leninsky Ave., Moscow 119991, Russia; 2 National Research Centre “Kurchatov Institute”, 1, Kurchatov Sq, Moscow 123182, Russia

**Keywords:** iron-based superconductors, IBS, 1144, microstructure, 2D pinning centers, HRTEM, magnetic superconductors

## Abstract

In the stoichiometric iron-based superconductor EuRbFe_4_As_4_, superconductivity coexists with a long-range magnetic ordering in Eu layers. Using high-resolution transmission electron microscopy (HRTEM), we observed an atomic structure of as-grown EuRbFe_4_As_4_ crystals. HRTEM shows that crystals have two-dimensional intrinsic nanoinclusions established to be the RbFe_2_As_2_ (122) phase with a volume fraction of ~5.6%. In contrast with the CaKFe_4_As_4_ compound, similar inclusions are not superconducting down to 2 K, and no second magnetization peak was observed in the magnetization measurements at low temperature with B ‖ ab. We show that the non-superconducting 122 phase nanoinclusions could act as 2D pinning centers.

## 1. Introduction

Nowadays, scientists all over the world are actively researching so-called magnetic superconductors in which superconductivity and ferromagnetism coexist simultaneously, which previously seemed completely impossible. Up to the present time, superconducting transition temperatures in magnetic superconductors were quite low and did not exceed 10 K [1,2,3]. With the 2008 discovery of iron-based superconductors where superconductivity exists in the FeAs planes, critical temperatures increased significantly from 26 up to 57 K [4,5,6].

**Figure 1:**
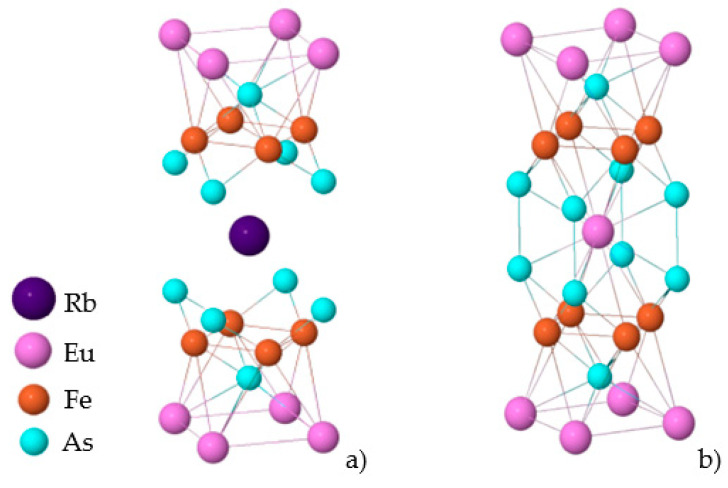
Crystal structure models of 1144 (**a**) and 122 (**b**).

A novel iron-based superconducting (SC) family, known as 1144-type, was discovered in 2016 [7,8]. The stoichiometric AeAFe_4_As_4_ compounds (Ae = Ca, Sr, Ba, Eu and A = K, Rb, Cs) with space symmetry group P4/mmm are formed by two alternating structures, AeFe_2_As_2_ and AFe_2_As_2_, see Figure 1a,b. Unlike the Ba-122 [9,10] system, the 1144-type does not form solid solutions because of the large difference in ionic radii between alkaline (A) and alkali-earth (Ae) atoms but fills the crystallographic positions alternately between the FeAs planes. Among the 1144 iron-based superconductors, the europium-containing compounds AEuFe_4_As_4_ (A = Rb, Cs) are the materials with coexistence between superconductivity and magnetism [11], with a magnetic transition temperature lower than the superconducting one (T_c_ = 35–37 K). Magnetic ordering of europium in the EuRbFe_4_As_4_ compound occurs at T_m_~15 K and has helical structure [12]. Despite the magnetic structure and the superconducting condensate being spatially separated from each other [13,14], magnetic interaction occurs between the Eu layers by superconducting ones. Such a pancake of alternating superconducting FeAs layers and magnetic Eu atoms is a natural analog of the layered superconductor–magnet (SC–M) heterostructure, but with self-assembly at the nanoscale, it opens up the prospects of Eu spin manipulation for electronics.

The value of the upper critical magnetic field H_c2_ in EuRbFe_4_As_4_ reaches 100 T [15]. Among other things, the EuRbFe_4_As_4_ compound has a surprisingly low superconducting anisotropy of ∼1.7 at low temperatures. The coherence lengths ξ_ab_ (in-plane) and ξ_c_ (out-of-plane) are 1.4 nm and 0.92 nm, respectively [16].

The growth of pure 1144 single crystals is not an easy task because of competition between the 1144 and the 122 phases during crystallization. While heating the initial components at temperature about T = 800 °C, two compounds, Ae-122 and A-122, are formed with the subsequent start of the 1144 phase formation at 900 °C [17]. According to the model given in [18], two conditions are necessary for the successful phase formation: lattice parameter matching and charge transfer between the blocks. For the 1144 phases, every fluctuation in synthesis leads to structural instabilities; therefore, obtaining a homogeneous 1144 phase is quite difficult due to the presence of a small fraction of 122 inclusions. During the EuRbFe_4_As_4_ synthesis, the appearance of both EuFe_2_As_2_ (Eu-122) and RbFe_2_As_2_ (Rb-122) impurity phases is possible. The EuFe_2_As_2_ compound has two phase transitions at about 190 K and 20 K [19]. The first one at T_0_ = 190 K is due to a spin density wave (SDW) along with a crystallographic phase transition from the tetragonal phase (I4/mmm) to an orthorhombic phase (Fmmm) and the AFM transition in the iron sublattice [20]. The phase transition at 20 K is caused by the AFM ordering of the Eu^2+^ moments. In Rb-122, temperature dependence of the resistivity behavior is the same as for a normal metal. The magnetic susceptibility, however, shows the broad peak around 80 K [21]. This feature has been assumed to be a disordered magnetism [22] or an orbital selective Mott insulator transition in the normal state [23] similar to KFe_2_As_2_.

Currently, the CaKFe_4_As_4_ compound microstructure has been studied in detail [24,25] with the use of HRTEM, where two-dimensional superconducting intergrowths of (Ca, K)Fe_2_As_2_ compatible with the appearance of second magnetization peak were found. However, there are no EuRbFe_4_As_4_ microstructure investigations explaining the vortex pinning properties. Thus, we study the single crystals of EuRbFe_4_As_4_ by HRTEM to find the correlation between the structure defects and SC properties.

## 2. Materials and Methods

The EuRbFe_4_As_4_ single crystals were prepared by the “self-flux” method in an RbAs flux. The preparation was carried out in a glove box in an argon atmosphere. High-purity metals Eu (Lanhit, 99.95%), Fe (ABCR, 99.98%), Rb (NZRM, 99.99%), and As (Lanhit, 99.9999%) were used to produce precursors EuAs, Fe_2_As, and RbAs. An excess of Rb was used to compensate Rb evaporation, and prolonged synthesis was performed to obtain up to the 100% of the RbAs phase. The obtained precursors were mixed in the required stoichiometric ratio with an addition of flux [RbAs] by the following reaction (1):

EuRbFe_4_As_4_ = EuAs + 2Fe_2_As + RbAs + 2[RbAs] (1)

**Figure 2:**
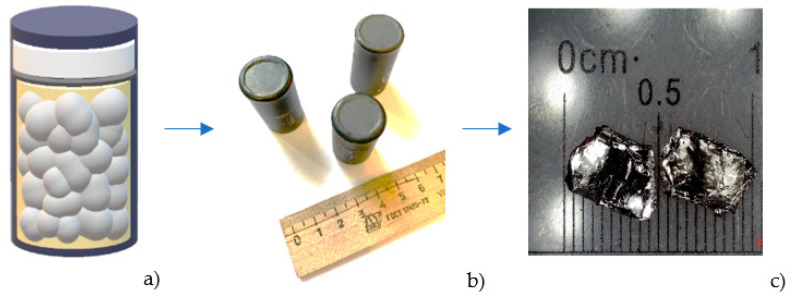
EuRbFe_4_As_4_ single crystals obtaining scheme: (**a**) reactants embedded in an alumina crucible and (**b**) welded in a niobium container, (**c**) EuRbFe_4_As_4_ single crystals.

Then, the reactants were placed in an alumina crucible, welded in a niobium container under residual argon pressure of 0.2 atm (Figure 2a,b), and subjected to a multi-stage heat treatment similar to our previous works with a FeAs flux [26,27]. After the synthesis, EuRbFe_4_As_4_ (EuRb-1144) crystals were collected from the crucible in an argon glove box and stored in it to avoid contact of the grown crystals with the air atmosphere and possible degradation. In our previous work [26], we showed the as-grown EuRb-1144 single crystals to be degraded after air exposure. The cleaved EuRbFe_4_As_4_ single crystals with a smooth and shiny surface are shown in Figure 2c.

Temperature dependences of the AC magnetic susceptibility and magnetic moment measurements in B ‖ ab and B ‖ c orientations up to 9 T were measured with a PPMS-9 Quantum Design system (San Diego, CA, USA). The AC magnetic susceptibility measurements were carried out with H_ac_ = 5 Oe. XRD (X-Ray diffraction) analysis was conducted with Rigaku Smartlab (Osaka, Japan) (in-plane *2θ/ω* scan) at Cu *Kα* with Ge-monochromator (220) × 2. The diffraction pattern was obtained in the range of *2θ* angles 5–60°.

The SEM (scanning electron microscopy) investigations were performed with a Helios NanoLab 600i scanning electron-ion microscope (Thermo Fisher Scientific, Waltham, MA, USA) equipped with EDS (energy-dispersive spectroscopy) analyzer (Ametek, Berwyn, PA, USA), Pt and W gas injection systems (GIS), and an OmniProbe 200 micromanipulator (Oxford Instruments, Abingdon, Oxfordshire, UK). For HRTEM studies, samples were prepared by a focused ion beam (FIB) technique. The samples were studied using a Titan 80–300 (Thermo Fisher Scientific, MA, USA) transmission (scanning) electron microscope with accelerating voltage of 300 kV with a beam probe size in the STEM mode of 0.8 A. The microscope is equipped with a sample spherical aberration corrector (Cs-corrector), a high-angle annular dark-field detector (HAADF), an X-Ray microanalysis system (Ametek (EDAX), PA, USA), and a characteristic electron energy loss spectroscopy system (Gatan (Ametek), Pleasanton, CA, USA). Image post-processing was performed using Digital Micrograph (Gatan (Ametek), CA, USA) and Tecnai Imaging and Analysis (Thermo Fisher Scientific, MA, USA) software.

## 3. Results and Discussion

**Figure 3:**
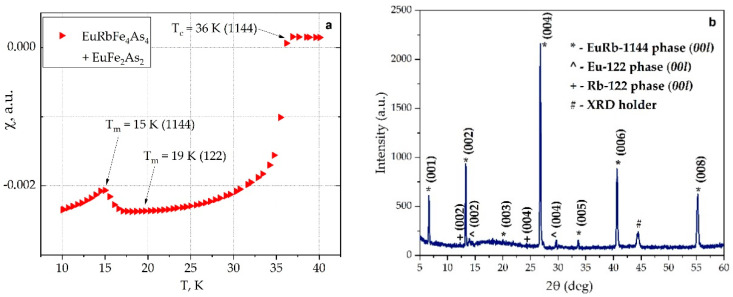
(**a**) Temperature dependence of the AC magnetic susceptibility of the EuRb-1144 sample (T_m_ ≈ 15 K, T_c_ ≈ 36 K) and non-superconducting trace inclusions of the Eu−122 phase (T_m_ ≈ 19 K); (**b**) XRD pattern of the EuRb-1144 crystal.

Before HRTEM investigations, we selected a sample with a negligible feature around 19 K in magnetic measurements associated with EuFe_2_As_2_ phase inclusions. To prevent the delamination of the single crystals, all necessary manipulations were performed in an argon glove box using parafilm or vacuum desiccator. Figure 3a shows temperature dependence of the AC susceptibility of EuRbFe_4_As_4_ single crystal selected for TEM investigations along the ab plane. Using the onset criteria, we found the critical temperature of the superconducting transition T_c_ (T_c_ = 36 K). The T_c_ value is similar to one presented in other papers [26,28]. The Eu^2+^ magnetic ordering of EuRb-1144 feature at T_m_ ≈ 15 K is shown in Figure 3a. In Figure 3b, one can see the XRD pattern of the EuRb-1144 crystal by in-plane *2θ/ω* scan. The pattern contains only reflections with *(00l)* orientation that demonstrate the monocrystallinity of the sample. In addition, a small amount of Rb-122 (see Rb-122 *(002)* and *(004)* reflections) and Eu-122 (see Eu-122 *(002)* and *(004)* reflections) phases were detected.

**Figure 4:**
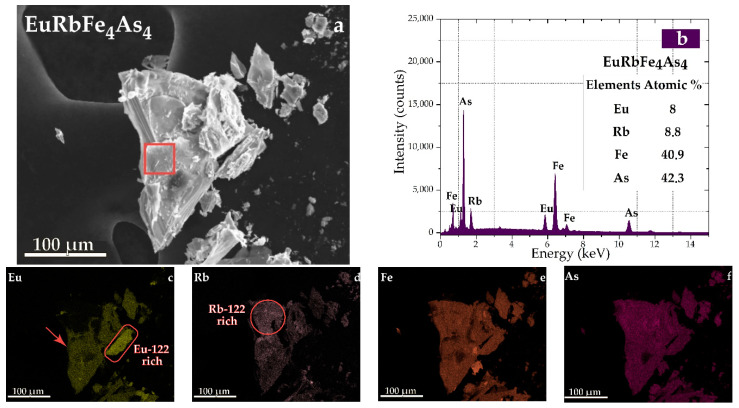
(**a**) SEM images of the EuRbFe_4_As_4_ single crystal in mapping mode; (**b**) EDS analysis spectra of the EuRbFe_4_As_4_; and maps of: (**c**) Rb; (**d**) Eu; (**e**) Fe; (**f**) As.

Then, part of the selected sample with a minimum inclusion of 122 phase was loaded into the sample exchange chamber, evacuated, and transferred to the microscope chamber, and elemental mapping of the surface of the EuRbFe_4_As_4_ single crystal was carried out by scanning electron microscopy, see Figure 4.

An energy dispersive (EDS) analysis confirms the presence of some areas with Eu-122 and Rb-122 phase predomination in the EuRb-1144 sample as shown in Figure 4c. We chose the single crystal part indicated by the red arrow (Figure 4c) for the lamella cutting, which had a required stoichiometric composition EuRbFe_4_As_4_ (Figure 4b). The lamella was cut along the c axis and investigated by a transmission electron microscope.

**Figure 5:**
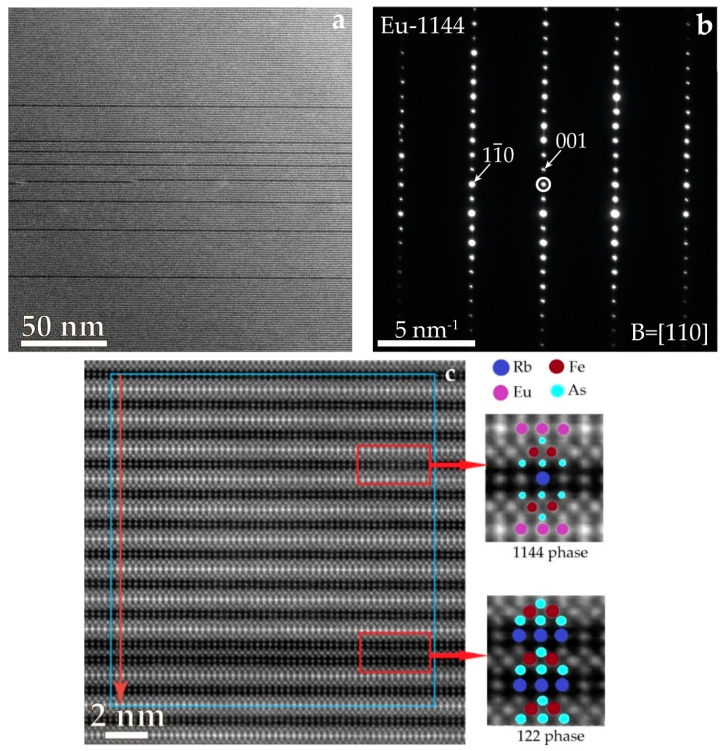

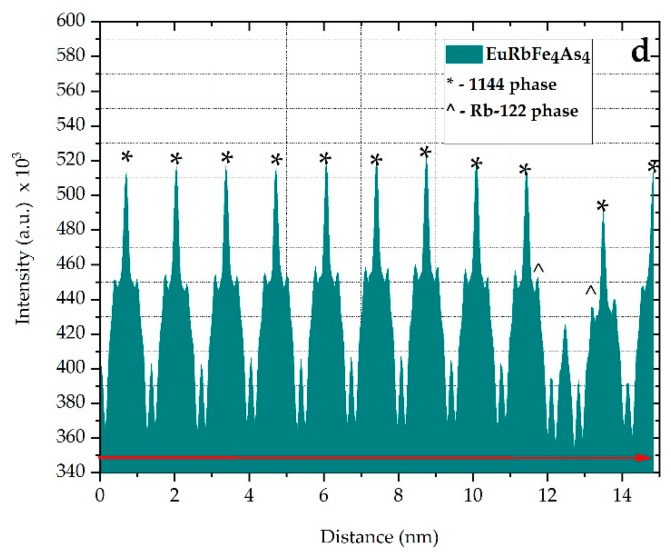
HAADF STEM images (**a**,**c**) of microstructures of the EuRbFe_4_As_4_ with black stripes of two-dimensional defects of the RbFe_2_As_2_, selected area diffraction pattern from EuRb−1144 phase (**b**); intensity profile (**d**); from the selected blue area (**c**).

Figure 5a,c present the HAADF STEM images of EuRbFe_4_As_4_ single crystal microstructures along the c plane. In the bulk of the EuRb-1144 sample, dark stripes of inclusions are observed, which are located along the ab plane. HAADF STEM images have Z-contrast; therefore, the dark stripes correspond to the absence of Eu atoms. A high-resolution image (Figure 5c) of defect and the corresponding intensity profile (Figure 5d) confirm this. The lattice parameters for the structural 122 defects were determined from the profile and found to be a, b = 0.38 nm, c = 1.45 nm, which is in a good agreement with the Rb-122 crystal structure data. The atomic lattice period for the EuRb-1144 phase was determined from the electron diffraction pattern, Figure 5b. The lattice parameter of EuRb-1144 is c = 1.319 nm and a, b = 0.388 nm, which complies well with the reference data [29].

The length of two-dimensional nanodefects along the ab plane is more than 200 nm, and the volume of inclusions is about 5.6% from the selected area 200 × 200 nm^2^ (Figure 5a). It should be noted that our sample was grown in the RbAs flux; thus, the Rb-122 intergrowths should dominate. Moreover, the electron diffraction pattern from the inclusion of Rb-122 cannot be obtained due to the small area. In the case of FeAs flux growth, the EuFe_2_As_2_ inclusions might be more frequent. Iyo et al. experimentally show a second magnetization peak (SMP) in CaK-1144 samples in the applied external magnetic field along ab direction [24]. According to the proposed model, the SMP was associated with superconducting nanoscale (Ca, K)Fe_2_As_2_ intergrowths inherent to a CaK-1144 single crystal along the ab plane. In our EuRbFe_4_As_4_ single crystal, the directly observed RbFe_2_As_2_ single nanolayers should not be superconducting down to 2.1–2.6 K [30,31]. The possible two-dimensional inclusions of EuFe_2_As_2_ (it was registered by magnetic susceptibility and XRD measurements) are well-known to be non-superconducting [32]. Thus, according to the abovementioned qualitative model, there should not be an SMP on the magnetic irreversibility loops above 2 K. To verify this statement, we measured the magnetic irreversibility loops at 2 and 4 K in fields up to 9 T in parallel and perpendicular field orientation to the ab plane.

**Figure 6:**
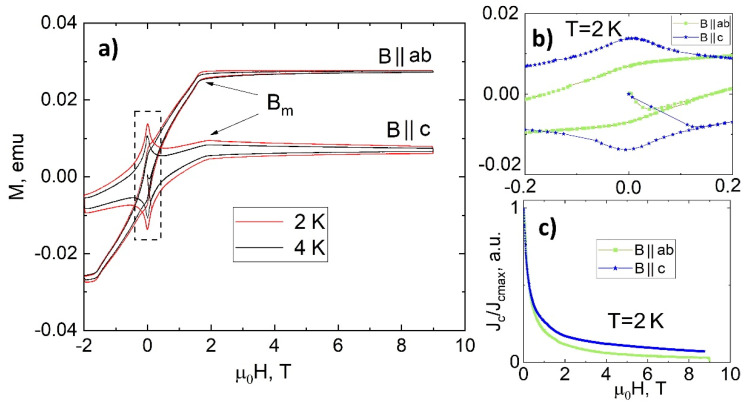
(**a**) Isothermal magnetization hysteresis data for EuRb−1144 as a function of magnetic field (up to 9 T) with B ‖ ab and B ‖ c at 2 and 4 K; (**b**) enlarged view of the central part of the hysteresis at 2 K (area indicated by the dashed line); (**c**) field dependence of J_c_/J_c_ max ∝ ∆M in the B ‖ ab and B ‖ c at 2 K for EuRb−1144 single crystal.

In Figure 6, one can find the magnetic irreversibility loops of investigated EuRbFe_4_As_4_ single crystal at T = 2 and 4 K with the B ‖ ab and B ‖ c, respectively. It is clearly seen that there are not any features in all cases with the exception of the kink of the loops attributed to magnetic nanoinclusions (Eu-122, iron atoms). The enlarged central part of the hysteresis loops at T = 2 K for both field directions presented in Figure 6b show a clear diamagnetic signal with increasing the magnetic field from B = 0, which point to domination of the superconducting signal. As shown recently, the loop width (∆M) value is weakly depend on magnetic impurities and on helical magnetic ordering in EuRb-1144 down to low temperatures [16,26]. Therefore, we may use Bean’s critical state model [33] without taking into account the contribution of the magnetic inclusions in order to investigate J_c_ curves behaviour. We calculated J_c_/J_c_ max(B) curves at T = 2 K (much lower than the magnetic transition), where J_c_ ∼ ∆M, and plotted in Figure 6c. One can clearly see the monotonous J_c_(B) dependence without any second magnetization peak in both field orientations. Similar to the CaKFe_4_As_4_ (CaK-1144) which shows significant SMP in B ‖ ab field orientation, in the EuRb-1144 system we found 122 phase monolayered nanoscale intergrowths (Rb-122) in the volume of the EuRb-1144 superconductor. However, the Rb-122 nanolayers seem to be non-superconducting down to 2 K and have no visible second magnetization peak appearance on the magnetic irreversibility loops. This experimentally confirms the assumption that the SMP was attributed to SC nanoscale intergrowths along the crystallographic ab plane. In our previous paper [26], we claimed the two-dimensional pinning predominance in the EuRbFe_4_As_4_ system with B ‖ ab. The direct HRTEM observation of Rb-122 nanoscale intergrowths support our suggestion about the predominance of two-dimensional pinning of the Abrikosov vortices in this system.

## 4. Conclusions

In our paper, we have investigated single crystals of the stoichiometric iron-based superconductor EuRbFe_4_As_4_ grown by the self-flux method. Using high-resolution transmission electron microscopy, we observed an atomic structure of as-grown EuRbFe_4_As_4_ single crystals. HRTEM shows that crystals have two-dimensional intrinsic nanoscale inclusions that are found to be RbFe_2_As_2_ (122) phase with a volume fraction of ~5.6%. We establish the correlation between the structure defects in the single crystals of EuRbFe_4_As_4_ and SC properties. This nanoinclusions are not superconducting, so there is no second magnetization peak in the EuRb-1144 compound (as opposed to CaK-1144 in the B ‖ ab). We found the defects of the non-superconducting Rb-122 phase in the external magnetic field B ‖ ab have a two-dimensional layered structure. We assume the Rb-122 nanoinclusions in the EuRbFe_4_As_4_ compound act as 2D pinning centers.

## Data Availability

The data that supports the findings in this study are available from the corresponding author upon reasonable request.
